# Psychometric properties of CVFQ7-BR-toxo to evaluate vision-related quality of life in children with congenital toxoplasmosis in Brazil

**DOI:** 10.5935/0004-2749.20220007

**Published:** 2022

**Authors:** Jacqueline Domingues Tibúrcio, Daniel Vitor Vasconcelos-Santos, Galton Carvalho Vasconcelos, Ericka Viana Machado Carellos, Roberta Maia de Castro Romanelli, Jose Nélio Januario, Gláucia Manzan Queiroz Andrade

**Affiliations:** 1 Departamento de Medicina, Universidade Federal de São João del-Rei; São João del-Rei, MG, Brazil; 2 Departamento de Oftalmologia e Otorrinolaringologia, Faculdade de Medicina da Universidade Federal de Minas Gerais, Belo Horizonte, MG, Brazil; 3 Hospital São Geraldo/Hospital das Clínicas da Universidade Federal de Minas Gerais, Belo Horizonte, MG, Brazil; 4 Departamento de Pediatria, Faculdade de Medicina da Universidade Federal de Minas Gerais, Belo Horizonte, MG, Brazil; 5 Hospital das Clínicas da Universidade Federal de Minas Gerais, Belo Horizonte, Minas Gerais, Brazil; 6 Departamento de Clínica Médica, Faculdade de Medicina, Universidade Federal de Minas Gerais, Belo Horizonte, MG, Brazil; 7 Núcleo de Ações em Pesquisa e Apoio Diagnóstico, Faculdade de Medicina, Universidade Federal de Minas Gerais, Belo Horizonte, MG, Brazil

**Keywords:** Quality of life, Low vision, Uveitis, Toxoplasmosis, congenital, Child, Qualidade de vida, Baixa visão, Uveíte, Toxoplasmose congênita, Criança

## Abstract

**Purpose:**

The high prevalence and severity of congenital toxoplasmosis in Brazil, with several affected individuals progressing to low vision, emphasize the importance of evaluating their quality of life. In this study, the Children’s Visual Function Questionnaire (CVFQ) was adapted to the sociocultural context of Brazilian children, and its psychometric properties were investigated for evaluating the vision-related quality of life of these individuals.

**Methods:**

This was a nested cross-sectional epidemiological study that prospectively monitored a cohort of 142 preschool children at a single referral university hospital in Belo Horizonte, Brazil. All children underwent complete ophthalmological examination, including visual acuity testing and binocular indirect ophthalmoscopy. Questionnaires were applied to their parents and caregivers to evaluate quality of life perception, as well as socioeconomic status of their families. Psychometric properties of the quality of life scale were evaluated by multivariate statistical analyses.

**Results:**

Adaptation to the Brazilian version of CVFQ-7 resulted in CVFQ-BR-toxo, a questionnaire for evaluating the perception of parents/caregivers about the vision-related quality of life of preschool children with congenital toxoplasmosis. The following six subscales were identified based on description, variability structure, and interpretation/grouping of items: general health, visual acuity, visual performance/functional vision, personal and social behavior, impact on family, and treatment. Children with low vision related to congenital toxoplasmosis had significantly lower scores for the following subscales: visual acuity (p=0.004), visual performance/functional vision (p=0.008), impact on family (p=0.001), and overall health (p=0.001).

**Conclusion:**

Psychometric properties were appropriate concerning the validity of the quality of life construct. CVFQ-BR-toxo could demonstrate the impact of vision impairment on families of children with congenital toxoplasmosis.

## Abstract (pt)

**Objetivo:**

A alta prevalência e gravidade da toxoplasmose congênita no Brasil, com muitos indivíduos afetados desenvolvendo baixa visão, reforça a importância da avaliação da sua qualidade de vida. Este estudo tem como objetivo adaptar o *Children’s Visual Function Questionnaire (CVFQ)* para a realidade sociocultural de crianças brasileiras e investigar suas propriedades psicométricas para avaliação da qualidade de vida relacionada à visão nesses indivíduos.

**Métodos:**

Estudo epidemiológico transversal aninhado de coorte de 142 crianças pré-escolares acompanhadas prospectivamente em hospital universitário de referência em Belo Horizonte, Brasil. Todas foram submetidos a exame oftalmológico completo, incluindo medida da acuidade visual e oftalmoscopia binocular indireta. Questionários foram aplicados aos pais e cuidadores, para avaliar a percepção da qualidade de vida, bem como o nível sócio-econômico das famílias. Análise estatística multivariada foi realizada para avaliar as propriedades psicométricas da escala de qualidade de vida.

**Resultados:**

Adaptações na versão brasileira do *Children’s Visual Function Questionnaire*-7 originaram o *Children’s Visual Function Questionnaire-*7-BR-toxo, um questionário para avaliar a percepção de pais/cuidadores sobre a qualidade de vida relacionada à visão de crianças pré-escolares com toxoplasmose congênita. Pela descrição, estrutura de variabilidade, e interpretação do agrupamento dos itens do questionário adaptado, identificaram-se seis subescalas: saúde geral, capacidade visual, desempenho visual/visão funcional, comportamento social e pessoal, impacto na família e tratamento. Crianças com baixa visão associada a toxoplasmose congênita tiveram escores mais baixos nas seguintes subescalas: acuidade visual (p=0,004), desempenho visual/visão funcional (p=0,008), impacto na família (p=0,001) e saúde geral (p=0,001).

**Conclusão:**

As propriedades psicométricas foram adequadas no tocante à validade do construto. O *Children’s Visual Function Questionnaire-*7-BR-toxo foi capaz de registrar o impacto da deficiência visual nas famílias de crianças com toxoplasmose congênita.

## INTRODUCTION

Congenital toxoplasmosis (CT) is an important disease in Brazil due to its highest prevalence rate compared with other countries in the world^(1)^. In the state of Minas Gerais, southeastern Brazil, CT is not only highly prevalent^(2,3)^ but also closely associated with lowest human development indices^(3)^.

Retinochoroiditis is a major clinical manifestation of CT^(4)^, and is responsible for causing more severe vision impairment in Brazilian children^(5,6)^. Early diagnosis and treatment of CT may improve the visual prognosis of infected children^(7,8)^. However, the impact of this disease on the quality of life (QOL) of individuals still remains largely unknown^(9)^.

Most of the instruments used to measure QOL have been developed in the USA and in Europe. Studies recommend that translated instruments should be evaluated for their equivalence of content and technique, internal consistency, and the manner in which they are organized and expressed for individuals of different cultural backgrounds. Quality is determined by the characteristics of the instrument, particularly its reliability and validity^(10,11)^.

Psychometrics implies the construction and validation of measurement instruments and evaluating whether these instruments are reliable and valid forms of measurement. In behavioral medicine, psychometrics is generally concerned with measuring an individual’s knowledge, ability, personality, and types of behaviors. In general, measurement is conducted in the form of a questionnaire, and questionnaires must be evaluated extensively before they can be stated to have excellent psychometric properties, i.e., a scale is both reliable and valid^(12,13)^.

The Children’s Visual Function Questionnaire (CVFQ) was developed to measure the impact of visual impairment on children and their families. It includes specific versions for the age groups below 3 years (CVFQ-3) and between 3 and 7 years (CVFQ-7)^(14)^. The Brazilian Portuguese version of CVFQ had its translation validated by a study conducted on children with congenital cataract^(15)^.

Considering the lack of validation studies of questionnaires of vision-related QOL in children with CT, we aimed to adapt the CVFQ-7 instrument and examine its psychometric properties for evaluating the vision-related QOL of these individuals.

## METHODS

This research was an observational nested cross-sectional epidemiological study. Institutional review board approval was obtained (no. CAAE/05040.0.203.000-11), and participants were included after their parents/caregivers signed the informed consent.

Individuals were recruited from a prospective population-based investigation on neonatal screening for CT, which had identified 190 infected neonates in Minas Gerais during 2006-2007^(4)^. A total of 155 children were still in the follow-up at our university hospital at the beginning of this study, thus being eligible for the study. Eight other children were referred by the pediatric infectious diseases division, resulting in a total of 163 children.

All children included in this study had CT defined according to the following criteria: anti-*Toxoplasma gondii* IgA and/or IgM in the first 6 months of life and/or persistent IgG at 12 months^(16)^. They were treated with sulfadiazine, pyrimethamine, and folinic acid for 12 months according to the international recommendations^(17)^, after which they made annual visits to the university hospital for pediatric, ophthalmological, and speech evaluation.

Children with delayed development, identified by Denver II screening test^(18)^, and those living in shelters, under the custody of child protective services, or with relatives under State custody were excluded. Those missing the eye examinations during the study period were also excluded.

Ophthalmic evaluation of the children consisted of medical history, external eye examination, refraction (retinoscopy), best-corrected visual acuity (BCVA), color vision and contrast sensitivity, ocular alignment/motility, confrontation visual field examination, slit-lamp biomicroscopy of the anterior segment, and dilated binocular indirect ophthalmoscopy.

Visual acuity was measured using ETDRS charts or Lea Symbols (LS) Test with backlighting, with identification of at least four of five optotypes. BCVA was registered for both eyes (OU), right eye (OD), and left eye (OS), and the result was expressed as a fraction. Visual loss was classified as low vision when BCVA <0.32 (20/63) or linear scale logMAR >0.5^(19)^.

The CVFQ instrument was applied to parents/caregivers to evaluate their perception on QOL related to their children’s visual function^(14)^. The CVFQ’s English language version is open access^(20)^, and its translation into Brazilian Portuguese has been validated^(15)^.

The CVFQ for the age group 3-7 years (CVFQ-7) contains 40 items, which are divided into the following six subscales: general health (1 item), general vision (2 items), competence (15 items), personality (9 items), family impact (7 items), and treatment (5 items), addressing different aspects of the quality of children’s visual function in a 5-point Likert scale. Responses are rated from 0 to 1 in 0.25 intervals (0, 0.25, 0.5, 0.75, and 1), with 1 indicating “best” and 0 indicating “worst” QOL. The global scale is obtained by averaging the scores of the items comprising the following subscales: competence, personality, family impact, and treatment. The items marked as “Does not apply to my child” or “My child is too young to attempt this” were excluded from this calculation.

The adaptation process of the Brazilian Portuguese version of CVFQ-7 to the context of children with CT in Minas Gerais was implemented during the regular meetings with specialists (pediatric infectious disease specialists, ophthalmologists, physical therapists, and occupational therapists) who provide care for children with visual impairment and with seven caregivers of children with CT and visual impairment who were born outside the period considered in this study. Caregivers evaluated the questionnaire regarding understanding, clarity, and difficulty in responding to each item.

In some cases, the group decided to include explanations of items in parentheses to facilitate understanding. The adapted questionnaire was termed CVFQ7-BR-toxo and is shown in the Appendix (online supplementary material).

After informed consente, the parents/caregivers of 142 children answered questions on their socioeconomic conditions and also responded to the questionnaires CVFQ7-BR-toxo and PedsQL^TM^ 4.0 Generic Core Scale^(21)^, the latter being a generic instrument for QOL assessment, which has been already validated in Brazil^(22)^.

Moreover, during the study, 29 randomly selected parents/caregivers were retested for CVFQ7-BR-toxo, 6 months apart (test-retest approach). All questionnaires were applied by the lead researcher.

Collected data were stored on the EpiData software version 3.1 (EpiData Association, Odense, Denmark) using the double typing technique. To evaluate data homogeneity, the coefficient of variation (CV) was used, which expresses the dispersion of data in relative terms by comparing standard deviation (*s*) to the mean (*χ*).

To examine the psychometric properties of CVFQ7- BR-toxo, we used multivariate statistics techniques^(23)^, including evaluation of the following aspects: 1) validity of QOL construct through exploratory factor analysis using the method of orthogonal rotation of varimax factors to describe the structure of variability, construct composition, suitability of the items to the factors (subscales), and their interpretation. For this purpose, we evaluated factor loading, which is the value of the correlation coefficient between each variable and its obtained factor, and the eigenvalue, which represents how much of the total variance is explained by each factor. Subscales were defined by interpretation of the underlying common dimension of factor loadings of items in each factor. In this process, each factor was initially presumed to represent a different dimension. However, the interpretation indicated that more than one factor was associated with the same underlying dimension or with a similar one, and the factors were combined to form a single subscale. When the item was present in more than one factor, it was allocated to the factor with the largest factor loading. Factors were composed of items with factor loading ≥0.45, as proposed in the original study^(14)^. The Kaiser-Meyer-Olkin Measure of Sampling Adequacy (KMO) test was used to evaluate the adequacy of the sample size to the factor model. Analysis of residues was conducted to evaluate the estimate of differences between correlations obtained in the original data and that reproduced by the adjusted factor model; 2) analysis of internal consistency through Cronbach’s alpha coefficient and 3) analysis of intraclass correlation and Wilcoxon test for comparing the median scores of the subscales on test-retest to evaluate reliability; 4) analysis of the discriminant construct validity by comparing the scores of the CVFQ7-BR-toxo subscales between patients identified with and without low vision through the Mann-Whitney test; and 5) analysis of the construct concurrent validity between the scores of the global scale of CVFQ7-BR-toxo and PedsQL^TM^ 4.0 Generic Core Scale by intraclass correlation.

## RESULTS

A total of 142 children aged 5-6 years (median age: 5 years) were included, with a predominance of boys (59.2%; 84). Most of them were cared by their parents during the day (64.8%; 92) and lived in an urban area (66.7%; 94) and in a house owned by their family (67.4%; 95). Parents/ caregivers had a low household income (0.5-2 minimum wages, equivalent to U$170-680 dollars monthly), and 40.4% (57) of them had only 4-7 years of education.

After ophthalmological examination, 27/142 children (19.0%) were diagnosed with LV, of whom 29.6% (8/27) wore glasses. Among the 142 children, 8.5% (12) had nystagmus and 32.4% (46) had strabismus. A total of 104 children (87.3%) had retinochoroidal scars consistent with toxoplasmosis; in 62.0% (88) of the children, these lesions were bilateral. Active retinochoroiditis was observed in 11.3% (16) of children during the study period. LV was identified in 83.3% (10/12) of children with nystagmus and in 34.7% (16/46) of children with strabismus.

Parents/caregivers answered the CVFQ7-BR-toxo in an average of 25 min. General health (Q1) was considered to be excellent by 18.5% of parents/caregivers (27), good and very good by 69.9% (102), and regular by 11.6% (17), with no significant differences when the frequencies of children with and without LV were compared.

Regarding visual ability (Q2), a significantly higher proportion of parents/caregivers classified the child’s vision as regular or bad (p=0.017) for children with LV (37.0%; 10/27) than parents/caregivers for children without LV (16.0%; 19/115).

Among the parents/caregivers, 40.8% (58) reported that their children had impaired vision in both eyes (Q3), which was less than what was found on the ophthalmic examination (69.7%; 99/142 children).

Analysis of the results of 11 items of CVFQ7-BR-toxo addressing how vision interferes with the child’s activities revealed the response “My child is too young to attempt this” in 4 items (36.4%), comprising 28.1%-84.9% of the answers to 4 items (Q19, Q22, Q24, and Q25).

The KMO test result was 0.74, indicating that the sample size was adequate to the factor model. The items were not indicative of multicollinearity, and 31% of the residues were >0.05. Initially, 11 factors were retained considering eigenvalues >1, representing 66.7% of the total explained variance. From the sixth factor onward, we found decreased impact of each factor on the total explained variance; therefore, we decided to extract six factors using the varimax rotation method. These corresponded to 46.3% of the total explained variance. Six of the 32 items (18.8%), Q4, Q6, Q26, Q29, Q30, and Q40, did not show factor loading in any of the six factors.

The six factors were grouped into four subscales, considering interpretation and description of variability structure. These subscales were named as visual performance/functional vision (factor 1, factor 5, and factor 6), family impact (factor 2), social and personal behavior (factor 3), and treatment (factor 4).

The item-overall scale correlations were significant (p<0.01 and >0.20). The subscales “visual performance/functional vision” and “family impact” were appropriate, with Cronbach’s alpha values of 0.75 and 0.77, respectively. The subscales “personal and social behavior” and “treatment” showed Cronbach’s alpha values <0.7 (0.68 and 0.61, respectively). All correlations between subscale scores and overall scale were significant (p<0.001), ranging from 0.42 to 0.74, indicating moderate to strong correlation between subscale scores and overall scale.

By comparing the scores of the parents/caregivers of children with and without low vision, we noticed a significant difference in the median scores of the subscales visual ability (p=0.004), visual performance/functional vision (p=0.008), and family impact (p=0.001), as well as a significant difference in overall QOL scores (p=0.001). Smaller median scores (Table 1) indicate a worse perception of eye health, greater family concern, and greater difficulty related to performance and skills that depended on visual function. There was no significant difference for the other subscales, namely general health, social and personal behavior, and treatment (p≥0.05).

**Table 1:** Scores reported by parents/caregivers of children with and without low vision

Subscale	Low vision		Mean			Standard deviation		Median	Variation coefficient	P value^a^
General health	No		0.64			0.23		0.75	0.36	0.490
	Yes		0.68			0.26		0.75	0.38	
Visual ability	No		0.80			0.17		0.8	0.21	0.004
	Yes		0.73			0.15		0.7	0.2	
Visual performance - functional vision	No		0.9			0.11		0.93	0.12	0.008
	Yes		0.82			0.16		0.86	0.19	
Family impact	No		0.85			0.2		0.92	0.23	0.001
	Yes		0.67			0.26		0.71	0.39	
Personal and social behavior	No		0.93			0.13		1.00	0.14	0.733
	Yes		0.92			0.13		0.95	0.14	
Treatment impact	No		0.67			0.27		0.75	0.39	0.244
	Yes		0.60			0.29		0.63	0.48	
QV global	No		0.86			0.11		0.88	0.12	0.001
	Yes		0.77			0.14		0.82	0.18	

a = Mann-Whitney test for median comparison: statistically significant for p<0.05.

The correlation between overall QOL scores of specific (CVFQ7-BR-toxo) and generic (PedsQL) instruments was moderate (*r*=0.58) and significant (p<0.001). The correlation among the subscale scores on test-retest was moderate to strong and significant (p<0.01). However, when the medians of these subscales were compared, no significant differences were detected (Table 2).

**Table 2:** Medians and correlation of the scores reported by parents/caregivers of children.

Subscale		CCI		Correlation IC (95%)			P value^a^	Test	Median Retest	P value^b^
General health		0.83		0.63 -	0.92		<0.001	0.50	0.5	0.613
Visual ability		0.69		0.33 -	0.85		0.002	0.90	0.8	0.096
Visual performance - functional vision		0.71		0.38 -	0.86		0.001	0.91	0.8	0.080
Family impact		0.84		0.65 -	0.92		<0.001	0.86	0.89	0.521
Personal and social behavior		0.95		0.9 -	0.98		<0.001	1.00	0.89	0.070
Treatment impact		0.63		0.22 -	0.83		0.005	0.63	0.75	0.616
QV global		0.83		0.64 -	0.92		<0.001	0.86	0.84	0.347

a = Intraclass correlation test: statistically significant for p<0.05.

b = Wilcoxon test for median comparison: statistically significant for p<0.05.

## DISCUSSION

To our knowledge, this is the first study to evaluate the vision-related QOL of Brazilian children with CT. Adaptation of CVFQ-7 to the context of parents/caregivers of children with CT allowed appropriate changes to the questionnaire without altering the meaning of each item.

Four items of the instrument were excluded from the analysis because of high frequency of the answer “My child is too young to attempt this.” These items indicated objects that children may not have easy access to, such as “bicycle, telephone, watch and coins.” Environmental stimulation for learning is critical for these activities, particularly in the setting of unfavorable socioeconomic conditions. Educational attainment of the parents is also believed to be an important factor in the development of the child because individuals with more school years have better vocabulary and capacity to promote child stimulation^(24)^. In the original CVFQ study, the researchers opted for exclusion of items when ≥95% of the answers were in the end of the scale or when ≥70% of the answers were “My child is too young to attempt this”^(14)^.

We decided to evaluate the structure of the model proposed by Felius et al.^(14)^ in the original CVFQ study and used by Lopes et al.^(15)^ to assess the vision-related QOL of children because of the cultural, social, and economic diversity in Brazil. Even when a preset model exists and the purpose is to test its application or consistency in a different population, it is highly recommended to evaluate the coherence of the structure as well as its interpretation^(25)^.

In the exploratory factor analysis, the results were similar to those of the original instrument^(14)^, indicating good suitability of the sample to the factor model and satisfactory residual analysis^(23,25)^. Six factors were extracted, corresponding to 46.3% of the total explained variance. Six items (18.8%) did not show factor loading in the extracted six factors. From a qualitative perspective, they can be justified for this population and corroborate the quantitative analysis. Items Q4 and Q6 are related to concern and time spent in the treatment of the child’s visual condition, which in this study often refers to check-ups with ophthalmologists on a yearly basis, or more frequently, in case of reactivation of retinochoroiditis. Item Q40 concerns family members/caregivers forgetting the child’s treatment. For this sample, monitoring service is vigilant and parents/caregivers are frequently requested to bring their children for regular follow-up. Item Q26 is related to social and cultural condition of the family, environmental stimulation, and access to objects. We observed that the parents/ caregivers in this study had low educational attainment (mean of 3.8 school years), 33.3% of them lived in rural areas (where access to culture/education is poor), and ≥80% of the children had attended school for the first time less than 6 months before the interview. Regarding items Q26 and Q29, we consider it important to specify the size, resolution, and contrast of images to better evaluate the influence of different objects such as books, television, and videos on children with low vision. Item Q30 is related to traveling. For the individuals examined in this study, this may be closely related to treatment, as the majority (87.9%) of them have to travel from their cities to the capital of our state for regular follow-up visits. Some parents/caregivers consider the trip as an opportunity to get out of their houses and visit relatives in the capital. However, others consider it as a physical and psychological strain as they may travel overnight and spend the entire day waiting for the appointment. These facts may affect their answer to this item.

As in the original study^(14)^, six factors were extracted, resulting in the following four subscales: visual performance/functional vision (factor 1, factor 5, and factor 6), family impact (factor 2), social and personal behavior (factor 3), and treatment (factor 4).

The 26 items with factor loading >0.45 comprised the subscales and were distributed according to the original study^(14)^, except for item Q15, which had a high factor loading in both the visual performance/functional vision subscale and the family impact subscale. Items Q15 and Q28 shared factor loadings with factors 1 and 2. As we observed a higher loading of Q15 in factor 2, it was allocated on the family impact subscale. This is justified because, in the absence of a reference distance to evaluate functional vision, it may contribute to family impact, unlike the results obtained by other studies in the literature^(14,15,26,27)^. In contrast, the much higher factor loading of Q28 in factor 1 can be interpreted considering its contribution to the evaluation of perception of the functional vision performance, as considered in other studies.

The homogeneity of measurements for the QOL construct in CVFQ7-BR-toxo was similar to that of the original study^(14)^ for visual performance (competence) and family impact subscales. Internal consistency was greater (Cronbach’s alpha >0.8) for the subscales personal and social behavior, as well as for treatment. For ability tests, a cutoff point of 0.7 is considered to be adequate^(28,29)^. Compared with the original study, we had a smaller number of items in all subscales.

A German study conducted on children with amblyopia and aphakia found results similar to those of our study^(26)^. In Brazil, a study on children with congenital cataracts revealed significant differences in all subscales^(15)^. It should be noted that the number of respondents in those studies was lower (approximately 50%) than that in our study.

Results of our study show that CVFQ7-BR-toxo can distinguish groups of individuals with and without low vision in different dimensions, thereby contributing to the assessment of vision-related QOL of children with CT. The moderate and significant correlation of overall QOL scores of specific (CVFQ7-BR-toxo) and generic (PedsQL) QOL assessment instruments suggests that these specific and generic instruments can be complementary for evaluating QOL perception.

CVFQ7-BR-toxo was reliable in terms of direct agreement (reproducibility), considering the lack of significant difference (p>0.05) in median scores between test-retest. In terms of relative agreement, the subscale scores on test-retest correlated significantly (p<0.001).

It is noteworthy that our study was conducted on a large population-based cohort of children with CT diagnosed and treated early since birth. To our knowledge, this was the first study in Brazil to evaluate the vision-related QOL of this population with a larger sample size than that of other studies using the CVFQ instrument^(15,26,27)^. Brazil is a country of abundant social, cultural, and economic diversity, which limits the extrapolation of our findings and necessitates adaptations in the instrument for application in significantly different contexts.

In conclusion, CVFQ7-BR-toxo demonstrated adequate psychometric properties concerning the validity of QOL construct. Adequate reproducibility and reliability suggest that it is useful to evaluate the vision-related QOL of children with CT. It was confirmed that the instrument can reveal the impact of visual impairment and visual performance on the families of children with CT.

## Appendix

### Items of the CVFQ instrument adapted to the Brazilian population of children with congenital toxoplasmosis (CVFQ7-BR-toxo)

The respondent must choose one of the following answer options by the type of nominal or ordinal scale according to: | Q | Nominal scale, | F | Frequency scale, | A | Agreement scale, | D | Difficulty scale. Some items have an additional answer: | NA |: Does not apply to my child and | TY | My child is too young to attempt this. The questionnaire items were preceded by a cover page with instructions and information about the purpose of this research.

1. In general, is your child’s overall health:

1. Excellent 2. Very Good 3. Good 4. Fair 5. Poor

2. At the present time, is your child’s eyesight when using both eyes:

1. Excellent 2. Good 3. Fair 4. Poor 5. Very Poor 6. Blind

3. If your child has an eyesight problem for only one eye, is your child’s eyesight in the affected eye:

1. Excellent 2. Good 3. Fair 4. Poor 5. Very Poor 6. Blind 7. Does not apply to my child

4. Do you worry about your child’s eyesight?

1. Never 2. Once in a while 3. Sometimes 4. Often 5. Always

5. How much time do you spend on caring for your child’s vision (such as eye doctor appointments, patching, eye drops, other medications, and therapy)?

1. Once a month or less (or never) 2. Once a week 3. Once a day 4. A few hours each day 5. Most of the day

6. Does the time you spend on your child’s vision (eye doctor appointments, patching, eye drops, other medications, and therapy) take away from time you would like to spend with your other children or husband/wife?

1. Never 2.Once in a while 3. Sometimes 4. Often 5. Always

### We would like to know how you feel about your child’s vision.

#### Please indicate how much you agree with each of the following statements:

1. Strongly Disagree 2. Disagree 3. Not Sure 4.Agree 5. Strongly Agree 6. Does not apply to my child

7. It bothers me when other people comment about my child’s vision or eyes when I take him/her somewhere.

8. My child feels different from other children.

9. My child is happy most of the time.

10. I notice other children looking at my child.

11. My child is teased because of his/her vision problems.

12. My child makes new friends easily.

13. My child is kind, affectionate.

14. My child gets along well with other children and friends.

### How does your child’s eyesight affect his/her activities?

#### Please indicate how much difficulty your child has with the following activities because of his/her vision condition:

1. No difficulty because of eyesight 2. A little difficulty because of eyesight 3. Moderate difficulty because of eyesight 4. Extreme difficult because of eyesight 5. Cannot do this at all because of eyesight 6. My child is too young to attempt this

15. My child can recognize faces (friends, relatives) across a room.

16. My child can get dressed by himself/herself.

17. My child can brush his/her teeth.

18. My child can wash his/her face.

19. My child can ride a bike (with or without training wheels)

20. My child can play sports or active games (such as soccer, tag, hide-and-seek, and games that involve running).

21. My child can pour liquid into a glass or cup.

22. My child can press phone keys.

23. My child helps with the chores (such as keeping toys tidy, putting dirty laundry in the hamper)

24. My child can tell the time.

25. My child can identify coins (by size or value).

### Please indicate how much you agree with each of the following statements:

1. Strongly Disagree 2. Disagree 3. Not Sure 4. Agree 5. Strongly Agree 6. Does not apply to my child.

26. My child enjoys looking at books.

27. My child’s eyesight makes it difficult for him/her to learn to walk, run, skip, or jump.

28. My child’s eyesight hinders his/her learning in everyday life or at school.

29. My child enjoys watching TV and videos or playing video games.

30. My child likes to travel with our family or to family’s and friends’ houses.

31. My child enjoys playing with others (sisters and brothers or friends).

32. My child enjoys drawing, painting, or other artistic activities.

33. My child’s eyesight makes it difficult for him/her to find something on a crowded shelf or in a closet.

### Please indicate how often this happens:

34. My child trips over curbs or steps.

1. Never 2. Once in a While 3. Sometimes 4. Often 5. Always 6. My child is too young to attempt this

### Questions about the treatment of your child’s eye condition.

35. Is your child currently being treated for his/her eye condition? (Treatment includes follow-up visits, eyeglasses, contact lenses, intraocular lenses, patching, eye drops, or other treatment).

1. Yes 2. No

### If your answer to questions 35 was YES, please answer the following questions:

1. Never 2. Once in a while 3. Sometimes 4. Often 5. Always

36. I have trouble taking my child to the doctor or applying treatment (for example, putting on an eye patch or glasses or giving eye drops or other medication).

37. My child is uncomfortable when taken to the doctor or treated (for example, while wearing a patch or glasses or when you put in eye drops).

38. My child is less active when taken to the doctor or treated (for example, when wearing a patch or glasses or when taking eye drops or medication).

39. I worry when my child refuses going into the doctor ’ s office or treatment (for example, pulls off the patch or glasses or squeezes eye shut when trying to put in eye drops).

40. I sometimes forget to treat my child.

CVFQ7-BR-toxo in Brazilian Portuguese

### CVFQ7-BR-Toxo

#### Children’s Visual Function Questionnaire

##### Questionário de Função Visual Infantil (Versão para crianças pré-escolares)

Os exames oculares não medem como a visão de uma (sua) criança afeta suas atividades diárias e o seu bem-estar geral. Nós estamos aplicando um questionário para medir como os problemas visuais afetam a (sua) criança na vida e como estes problemas visuais afetam suas famílias.

Nós não daremos qualquer informação sobre você ou seu(ua) filho(a) para qualquer outra pessoa.

##### Instruções

Por favor, leia ou ouça cada pergunta cuidadosamente;

É importante que você responda a todas as questões;

Tente optar por uma única resposta para cada pergunta;

Se seu filho usa óculos responda considerando esta condição;

Se seu(ua) filho(a) usa óculos ou oclusão (tampão), tente pensar em suas situações típicas do dia-a-dia enquanto responde às questões.

##### Por favor, responda às questões a seguir sobre a saúde e a visão do seu(ua) filho(a):

1. Em geral, a saúde geral de seu(ua) filho(a) é:

1. ( ) Excelente 2. ( ) Muito Boa 3. ( ) Boa 4. ( ) Razoável 5. ( ) Ruim

2. No momento, a visão de seu(ua) filho(a) quando está com os dois olhos abertos é:

1. ( ) Excelente 2. ( ) Boa 3. ( ) Razoável 4. ( ) Ruim 5. ( ) Muito Ruim 6. ( ) Cego(a)

3. Se o(a) seu(ua) filho(a) tem problema visual somente em um olho, a visão de seu(ua) filho(a) no olho com problema é:

1. ( ) Excelente 2. ( ) Boa 3. ( ) Razoável 4. ( ) Ruim 5. ( ) Muito Ruim 6. ( ) Cego(a) 7. ( ) Não se aplica a(o) meu(inha) filho(a)

4. Você se preocupa com a visão de seu(ua) filho(a)?

1. ( ) Nunca 2. ( ) Raramente 3. ( ) Às vezes 4. ( ) Frequentemente 5. ( ) Sempre

5. Quanto tempo você gasta com cuidados relacionados à visão de seu filho? (por exemplo consultas com oftalmologista, oclusão ou tampão, colírios, outros medicamentos, terapias)

1. ( ) Uma vez ao ano 2. ( ) Uma vez ao mês 3. ( ) Mais de uma vez por semana 4. ( ) Uma vez por dia 5. ( ) A maior parte do dia

6. O tempo que você gasta com a visão de seu(ua) filho(a) toma o tempo que você gostaria de gastar com seus outros filhos ou marido/esposa? (consultas com o oftalmologista, terapias, oclusão, colírios, outros medicamentos)

1. ( ) Nunca 2. ( ) Raramente 3. ( ) Às vezes 4. ( ) Frequentemente 5. ( ) Sempre

##### Nós gostaríamos de saber como você se sente sobre a visão de seu(ua) filho(a).

###### Por favor, indique o quanto você concorda com as afirmações a seguir:

7. Me incomoda quando outras pessoas comentam sobre a visão ou os olhos de meu(inha) filho(a) quando o(a) levo a algum lugar.

1. ( ) Discordo plenamente 2. ( ) Discordo 3. ( ) Não estou certo 4. ( ) Concordo 5. ( ) Concordo plenamente 6. ( ) Não se aplica a meu(inha) filho(a)

8. Meu(inha) filho(a) se sente diferente das outras crianças.

1. ( ) Discordo plenamente 2. ( ) Discordo 3. ( ) Não estou certo 4. ( ) Concordo 5. ( ) Concordo plenamente 6. ( ) Não se aplica a meu(inha) filho(a)

9. Meu(inha) filho(a) é feliz a maior parte do tempo.

1. ( ) Discordo plenamente 2. ( ) Discordo 3. ( ) Não estou certo 4. ( ) Concordo 5. ( ) Concordo plenamente 6. ( ) Não se aplica a meu(inha) filho(a)

10. Eu noto que as outras crianças reparam/olham o(a) meu(inha) filho(a).

1. ( ) Discordo plenamente 2. ( ) Discordo 3. ( ) Não estou certo 4. ( ) Concordo 5. ( ) Concordo plenamente 6. ( ) Não se aplica a meu(inha) filho(a)

11. Meu(inha) filho(a) sofre gozação por causa de seu problema visual.

1. ( ) Discordo plenamente 2. ( ) Discordo 3. ( ) Não estou certo 4. ( ) Concordo 5. ( ) Concordo plenamente 6. ( ) Não se aplica a meu(inha) filho(a)

12. Meu(inha) filho(a) faz novos amigos com facilidade.

1. ( ) Discordo plenamente 2. ( ) Discordo 3. ( ) Não estou certo 4. ( ) Concordo 5. ( ) Concordo plenamente 6. ( ) Não se aplica a meu(inha) filho(a)

13. Meu(inha) filho(a) é carinhoso(a), afetuoso(a).

1. ( ) Discordo plenamente 2. ( ) Discordo 3. ( ) Não estou certo 4. ( ) Concordo 5. ( ) Concordo plenamente 6. ( ) Não se aplica a meu(inha) filho(a)

14. Meu(inha) filho(a) convive bem com outras crianças e amigos.

1. ( ) Discordo plenamente 2. ( ) Discordo 3. ( ) Não estou certo 4. ( ) Concordo 5. ( ) Concordo plenamente 6. ( ) Não se aplica a meu(inha) filho(a)

##### Como a visão de seu(ua) filho(a) interfere nas atividades dele(a)?

###### Por favor, indique o quanto o problema na visão de seu(ua) filho(a) dificulta a prática das atividades:

15. Meu(inha) filho(a) consegue reconhecer rostos (amigos, parentes) do outro lado da sala.

1. ( ) Sem dificuldade por causa da visão 2. ( ) Um pouco de dificuldade por causa da visão

3. ( ) Dificuldade moderada por causa da visão 4. ( ) Dificuldade extrema por causa da visão

5. ( ) Não consegue fazer por causa da visão 6. ( ) Meu(inha) filho(a) é muito pequeno(a) para tentar isso

16. Meu(inha) filho(a) consegue se vestir sozinho(a).

1. ( ) Sem dificuldade por causa da visão 2. ( ) Um pouco de dificuldade por causa da visão

3. ( ) Dificuldade moderada por causa da visão 4. ( ) Dificuldade extrema por causa da visão

5. ( ) Não consegue fazer por causa da visão 6. ( ) Meu(inha) filho(a) é muito pequeno(a) para tentar isso

17. Meu(inha) filho(a) consegue escovar seus dentes.

1. ( ) Sem dificuldade por causa da visão 2. ( ) Um pouco de dificuldade por causa da visão

3. ( ) Dificuldade moderada por causa da visão 4. ( ) Dificuldade extrema por causa da visão

5. ( ) Não consegue fazer por causa da visão 6. ( ) Meu(inha) filho(a) é muito pequeno(a) para tentar isso

18. Meu(inha) filho(a) consegue lavar seu roso.

1. ( ) Sem dificuldade por causa da visão 2. ( ) Um pouco de dificuldade por causa da visão

3. ( ) Dificuldade moderada por causa da visão 4. ( ) Dificuldade extrema por causa da visão

5. ( ) Não consegue fazer por causa da visão 6. ( ) Meu(inha) filho(a) é muito pequeno(a) para tentar isso

19. Meu(inha) filho(a) consegue andar de bicicleta (com ou sem rodinha)

1. ( ) Sem dificuldade por causa da visão 2. ( ) Um pouco de dificuldade por causa da visão

3. ( ) Dificuldade moderada por causa da visão 4. ( ) Dificuldade extrema por causa da visão

5. ( ) Não consegue fazer por causa da visão 6. ( ) Meu(inha) filho(a) é muito pequeno(a) para tentar isso

20. Meu(inha) filho(a) consegue praticar esportes ou jogos ativos (por exemplo: jogar bola, brincadeiras de correr, pega-pega e pique - esconde).

1. ( ) Sem dificuldade por causa da visão 2. ( ) Um pouco de dificuldade por causa da visão

3. ( ) Dificuldade moderada por causa da visão 4. ( ) Dificuldade extrema por causa da visão

5. ( ) Não consegue fazer por causa da visão 6. ( ) Meu(inha) filho(a) é muito pequeno(a) para tentar isso

21. Meu(inha) filho(a) consegue despejar líquido num copo ou xícara.

1. ( ) Sem dificuldade por causa da visão 2. ( ) Um pouco de dificuldade por causa da visão

3. ( ) Dificuldade moderada por causa da visão 4. ( ) Dificuldade extrema por causa da visão

5. ( ) Não consegue fazer por causa da visão 6. ( ) Meu(inha) filho(a) é muito pequeno(a) para tentar isso

22. Meu filho consegue teclar no telefone.

1. ( ) Sem dificuldade por causa da visão 2. ( ) Um pouco de dificuldade por causa da visão

3. ( ) Dificuldade moderada por causa da visão 4. ( ) Dificuldade extrema por causa da visão

5. ( ) Não consegue fazer por causa da visão 6. ( ) Meu(inha) filho(a) é muito pequeno(a) para tentar isso

23. Meu(inha) filho(a) ajuda com os afazeres de casa (por exemplo guardar os brinquedo, colocar a roupa suja no cesto para lavar)

1. ( ) Sem dificuldade por causa da visão 2. ( ) Um pouco de dificuldade por causa da visão

3. ( ) Dificuldade moderada por causa da visão 4. ( ) Dificuldade extrema por causa da visão

5. ( ) Não consegue fazer por causa da visão 6. ( ) Meu(inha) filho(a) é muito pequeno(a) para tentar isso

24. Meu(inha) filho(a) consegue falar que horas são.

1. ( ) Sem dificuldade por causa da visão 2. ( ) Um pouco de dificuldade por causa da visão

3. ( ) Dificuldade moderada por causa da visão 4. ( ) Dificuldade extrema por causa da visão

5. ( ) Não consegue fazer por causa da visão 6. ( ) Meu(inha) filho(a) é muito pequeno(a) para tentar isso

25. Meu(inha) filho(a) consegue identificar moedas (pelo tamanho ou valor).

1. ( ) Sem dificuldade por causa da visão 2. ( ) Um pouco de dificuldade por causa da visão

3. ( ) Dificuldade moderada por causa da visão 4. ( ) Dificuldade extrema por causa da visão

5. ( ) Não consegue fazer por causa da visão 6. ( ) Meu(inha) filho(a) é muito pequeno(a) para tentar isso

##### Como a visão do seu filho interfere nas atividades dele?

###### Por favor, indique o quanto você concorda com as afirmações abaixo:

26. Meu(inha) filho(a) gosta de ver livros.

1. ( ) Discordo plenamente 2. ( ) Discordo 3. ( ) Não estou certo 4. ( ) Concordo 5. ( ) Concordo plenamente 6. ( ) Não se aplica a meu(inha) filho(a)

27. A visão de meu(inha) filho(a) dificultou seu aprendizado para andar, correr, saltar ou pular.

1. ( ) Discordo plenamente 2. ( ) Discordo 3. ( ) Não estou certo 4. ( ) Concordo 5. ( ) Concordo plenamente 6. ( ) Não se aplica a meu(inha) filho(a)

28. A visão de meu(inha) filho(a) atrapalha seu aprendizado no dia-a-dia ou na escola.

1. ( ) Discordo plenamente 2. ( ) Discordo 3. ( ) Não estou certo 4. ( ) Concordo 5. ( ) Concordo plenamente 6. ( ) Não se aplica a meu(inha) filho(a)

29. Meu(inha) filho(a) gosta de ver TV, vídeos, ou de jogar videogames.

1. ( ) Discordo plenamente 2. ( ) Discordo 3. ( ) Não estou certo 4. ( ) Concordo 5. ( ) Concordo plenamente 6. ( ) Não se aplica a meu(inha) filho(a)

30. Meu(inha) filho(a) gosta de viajar com a família ou para a casa de familiares e amigos.

1. ( ) Discordo plenamente 2. ( ) Discordo 3. ( ) Não estou certo 4. ( ) Concordo 5. ( ) Concordo plenamente 6. ( ) Não se aplica a meu(inha) filho(a)

31. Meu(inha) filho(a) gosta de brincar com outras crianças (irmãos ou amigos).

1. ( ) Discordo plenamente 2. ( ) Discordo 3. ( ) Não estou certo 4. ( ) Concordo 5. ( ) Concordo plenamente 6. ( ) Não se aplica a meu(inha) filho(a)

32. Meu(inha) filho(a) gosta de desenhar, pintar ou de outras atividades de artes.

1. ( ) Discordo plenamente 2. ( ) Discordo 3. ( ) Não estou certo 4. ( ) Concordo 5. ( ) Concordo plenamente 6. ( ) Não se aplica a meu(inha) filho(a)

33. A visão de meu(inha) filho(a) dificulta que ele(a) encontre algo em uma prateleira ou em um armário.

1. ( ) Discordo plenamente 2. ( ) Discordo 3. ( ) Não estou certo 4. ( ) Concordo 5. ( ) Concordo plenamente 6. ( ) Não se aplica a meu(inha) filho(a)

##### Por favor, indique com que frequência acontece:

34. Meu(inha) filho(a) tropeça em degraus ou no meio fio.

1. ( ) Nunca 2. ( ) Raramente 3. ( ) Às vezes 4. ( ) Frequentemente 5. ( ) Sempre 6. Meu(inha) filho(a) é muito pequeno(a) para tentar isso

#### Perguntas sobre o tratamento da condição ocular de seu(ua) fiho(a).

Seu filho está realizando tratamento para a condição visual dele (por exemplo, consultas de acompanhamento, uso óculos, lente de contato, lente intraocular, oclusão (tampão), colírios ou outro tipo de tratamento)?

Por favor, circule um:

#### SIM / NÃO

##### Se responder NÃO para a questão 35, siga para a próxima página.

###### Se responder SIM para a questão 35, por favor, responda as questões a seguir:

36. Eu tenho problema para levar meu filho (a) à consulta ou para aplicar o tratamento (por exemplo, colocar os óculos ou tampão, colírio ou outra medicação).

1. ( ) Nunca 2. ( ) Raramente 3. ( ) Às vezes 4. ( ) Frequentemente 5. ( ) Sempre

37. Meu(inha) filho(a) fica incomodado(a) quando é levado à consulta ou tratado(a) (por exemplo, quando usa óculos ou tampão ou quando recebe colírios).

1. ( ) Nunca 2. ( ) Raramente 3. ( ) Às vezes 4. ( ) Frequentemente 5. ( ) Sempre

38. Meu(inha) filho(a) é menos ativo(a) quando é levado à consulta ou quando tratado(a) (por exemplo, quando usa óculos ou tampão, ou quando recebe colírios ou outras medicações, consulta).

1. ( ) Nunca 2. ( ) Raramente 3. ( ) Às vezes 4. ( ) Frequentemente 5. ( ) Sempre

39. Eu me preocupo quando meu(inha) filho(a) recusa ser atendido pelo médico quando levado à consulta ou recusa o tratamento (por exemplo tira os óculos ou tampão, ou fecha os olhos na hora de colocar o colírio).

1. ( ) Nunca 2. ( ) Raramente 3. ( ) Às vezes 4. ( ) Frequentemente 5. ( ) Sempre

40. Eu algumas vezes esqueço à consulta ou o tratamento do(a) meu(inha) filho(a).

1. ( ) Nunca 2. ( ) Raramente 3. ( ) Às vezes 4. ( ) Frequentemente 5. ( ) Sempre
